# Resilience in Recovery: The Interplay of Coping Strategies and Psychological Factors Among Breast Cancer Survivors

**DOI:** 10.14740/wjon2822

**Published:** 2026-09-04

**Authors:** Maryam Hasan, Magda Bayoumi, Leena Khonji

**Affiliations:** a Nursing Department, College of Health and Sport Sciences, University of Bahrain, Zallaq, Kingdom of Bahrain

**Keywords:** Bahrain, Breast cancer survivors, Brief-COPE, Coping strategies, DASS-10, Distress, Oncology nursing, Psychological well-being

## Abstract

**Background:**

Breast cancer (BC) survivorship is associated with significant psychological challenges, including anxiety, stress, and depression. Coping strategies (C-Strats) play a crucial role in influencing psychological well-being (Psych-WB) among survivors, but few studies have explored interconnected factors among BC surviving women in Bahrain, a disadvantaged population. This study thus examines the links between psychological coping and well-being solutions as deployed among women after BC in the Kingdom of Bahrain and to explore variations according to selected demographic characteristics.

**Methods:**

A descriptive cross-sectional design was employed. A total of 104 BC survivors attending oncology clinics in governmental hospitals were recruited using convenience sampling. Data were collected using a demographic questionnaire, the Brief Coping Orientation to Problems Experienced Scale, and the Depression Anxiety Stress Scale. Descriptive and inferential statistics, including *t*-tests, analysis of variance, and Pearson correlation, were performed using SPSS version 28.

**Results:**

Participants demonstrated moderate use of C-Strats, with higher reliance on problem-focused and “emotion-focused coping,” particularly religious coping, and lower use of “avoidant coping.” Psych-WB scores indicated moderate levels of anxiety, stress, and depression. C-Strats did not differ significantly across demographic variables (P > 0.05). However, psychological distress was significantly associated with age (P = 0.035), with younger survivors reporting higher distress levels. A statistically significant moderate negative correlation was found between C-Strats and Psych-WB (r = −0.501, P < 0.001), indicating that higher coping ability was associated with lower psychological distress.

**Conclusion:**

The findings highlight the protective role of C-Strats in reducing psychological distress among BC survivors. Interventions aimed at enhancing adaptive coping, particularly among younger survivors, are recommended to improve Psych-WB.

## Introduction

Breast cancer (BC) has long been acknowledged as one of the most pressing global public health challenges, being the most diagnosed cancer among women worldwide. In 2022, an estimated 2.3 million women were diagnosed with BC globally, accounting for approximately 670,000 deaths, highlighting both the substantial burden of the disease and disparities in outcomes across different regions. Globally, BC accounts for nearly 12% of all new cancer cases in women [1]. Trends over the past decade indicated a modest but steady rise in BC incidence in many countries, including high-income nations.

In the United States, the incidence of invasive BC continues to increase by approximately 1% per year (1.4% among women under 50 years of age), while mortality rates have declined. This is largely due to advances in screening, early detection, and treatment, yet holistic discrepancies affect outcomes, as evident in racial disparities. For instance, Black women continued to experience higher mortality (despite having lower incidence) than White women. Globally, mortality reductions were more evident in higher-income countries, where access to early detection and effective therapies was well established [2], including more holistic and spiritual approaches to care and coping [3]; this is encompassed in the study instrument’s Brief Coping Orientation to Problems Experienced (Brief-COPE) instrument, as described below.

Conversely, many low- and middle-income countries faced rising incidence rates without corresponding improvements in survival, primarily due to limited health infrastructure, delayed diagnoses, and inadequate access to quality care [4].

In the Eastern Mediterranean Region and Gulf Cooperation Council (GCC) countries, BC constitutes a major proportion of the female cancer burden. In 2020, GCC countries reported approximately 42,475 new cancer cases, and female BC accounted for 16% of these cases. It is projected that cancer incidence and mortality in the GCC may double by 2040 (from 2020 levels) if current trends continue [4].

In Bahrain, BC was the most diagnosed cancer among women, representing a significant public health concern. In 2022, it accounted for 324 new female cancer cases, or 44.4% of all new female cancer diagnoses in the country, emphasizing its predominant role in the national female cancer profile. Historically, the “age-standardized incidence rate” (ASIR) in Bahrain was relatively high compared to neighboring countries. Between 2000 and 2010, ASIR declined from 58.2 to 44.4 per 100,000, with an average of 52.3 per 100,000 over that period. During this decade, only 12.7% of cases were detected through screening, and the mean age at diagnosis was 50.9 years [5].

BC, beyond being a physical health issue, entails profound psychological and emotional strains on survivors, including fear of recurrence, changes in body image, and uncertainty about life after treatment. These challenges lead to elevated anxiety, depression, and reduced quality of life (QoL), highlighting the critical need to address the psychological well-being (Psych-WB) of survivors [6].

The journey of BC survivors was marked by numerous physical, emotional, and psychological challenges. Survivors often face long-term effects of treatment, changes in body image, fatigue, and fear of recurrence, which significantly undermine their QoL. Coping strategies (C-Strats) are highly important, enabling survivors to manage stress, adapt to their changing circumstances, and maintain a sense of control. Effective coping mechanisms, such as problem-solving, positive reframing, and seeking social support, are linked with better psychological outcomes [7, 8].

Adaptive C-Strats, including problem-solving, emotional expression, and seeking help, allow survivors to effectively manage stress, whereas maladaptive coping often worsens psychological outcomes. Spirituality and self-compassion provide additional psychological resources, offering meaning, hope, and emotional strength during the survivorship journey [9].

To deal with cancer and survivorship stressors, patients adopt numerous strategies to cope, ranging from adaptive, healthcare professional-mediated programs to improve self-efficacy and self-care (and support-seeking behavior), to maladaptive *ad hoc* solutions developed autonomously (e.g., drinking alcohol or denial). Previous studies found that C-Strats like fatalism and helplessness were linked to higher levels of psychological distress among BC survivors [10, 11].

### Aim

This research aims to explore the interconnected links between access to social support, strategies for C-Strats, spirituality, and Psych-WB among survivors of BC (hereinafter “survivors”) in the Kingdom of Bahrain.

## Materials and Methods

A descriptive cross-sectional design was employed to examine the experiences of survivors. This design was chosen to evaluate C-Strats and Psych-WB at a single point in time, as it provides an efficient method for capturing these variables [12–14]. Data were collected from women diagnosed as survivors.

### Study setting

The setting for this study was primarily the oncology clinics of governmental hospitals, which served as a key point of care for patients diagnosed with BC. These clinics were not just medical facilities for treatment but also centers where patients received chemotherapy, follow-up care, and appointments, including psychological support and counseling. The choice of oncology clinics in governmental hospitals as the study setting was strategic, as these centers treat a diverse range of BC survivors (and current BC patients) related to demographics, disease severity, and treatment stages.

### Population

The population for this study included female survivors who attended oncology clinics at governmental hospitals in Bahrain. These participants were selected due to their accessibility and their relevance to the research objectives. Survivors were defined as women who had been diagnosed with BC and had undergone treatment, including mastectomy, within the past 5 years. This population (n = 120) was particularly significant as it represented individuals actively engaged in healthcare services and likely encountering the physical, psychological, and emotional challenges associated with survivorship. Eligible participants were female patients aged 20 years and above, capable of reading and understanding Arabic, and regularly attending oncology clinics or follow-up appointments for treatment.

### Sample size

Based on calculations using the Epi Info program version 10, the sample size for this study was set at a minimum of 91 participants. This size was determined using parameters such as a population size of 120 (the number of patients at the studied site), a 95% confidence level, a 50% expected frequency, and a 5% margin of error. The outcome variable considered for the sample size estimation was prevalence of psychological distress or QoL score among survivors. The chosen sample size was considered sufficient to provide reliable and statistically significant data, while also being manageable for in-depth analysis. During the research period (from June 2023 to December 2023), 104 out of 120 eligible patients expressed their willingness to participate in the study.

### Criteria for sample selection

The criteria for selecting participants were designed to ensure that the sample was sufficient to identify trends among the general service user group of survivors attending oncology clinics in governmental hospitals. The inclusion and exclusion criteria applied during participant selection are as shown in Table 1.

**Table 1:** Participants’ Inclusion and Exclusion Criteria

Inclusion criteria	Exclusion criteria
Female patients	Patients aged under 20 years
Patients aged 20 years and above	Patients with a history of cognitive impairment or mental illness that may interfere with their ability to complete the questionnaires
Diagnosed with breast cancer, having undergone mastectomy within the past 5 years	Women diagnosed with advanced or terminal breast cancer
Ability to read and understand Arabic	Patients with severe comorbidities that could limit participation
Regular attendance at oncology clinics or follow-up for treatment	Patients currently undergoing palliative care
	Women who are unwilling or unable to provide informed consent
	Patients receiving ongoing psychiatric treatment, including medication for sleep disorders or severe depressive episodes, that may influence psychological responses
	Patients participating in other conflicting clinical studies

Source: Authors’ original creation.

### Measurement tools

Three tools were used to collect the necessary data, as described below. The empirical questionnaires were validated by the original authors [15, 16].

#### Tool I: sociodemographic characteristics

A structured tool was used to collect demographic and clinical data, including age, education level, marital status, employment status, and comorbidities. Clinical details included BC stage, type of mastectomy surgery (e.g., unilateral, bilateral, partial), and any additional BC-related procedures specified by participants.

#### Tool II: Brief-COPE Self-Reported Questionnaire

##### 1) Description

The Brief-COPE Scale, developed by Carver (1997) [15], consists of 28 items designed to assess how individuals cope with stress. Each item is assessed as per a four-point Likert scale (e.g., with responses ranging through 1 = “not at all,” 2 = “sometimes,” 3 = “medium,” and 4 = “often”). Fourteen subscales are encompassed by the scale, each comprising two items, with possible subscale scores ranging from 2 to 8 points. A higher score on a subscale indicated greater use of that particular C-Strat. The Brief-COPE items were categorized according to triune domains of coping: “problem-focused coping,” “emotion-focused coping,” and “avoidant coping.”

##### 2) “Problem-focused coping” domain (items 2, 7, 10, 12, 14, 17, 23, and 25)

This domain reflected active and solution-oriented efforts to manage stressful situations. It included four subscales:

- Active coping (items 2 and 7)
- Use of informational support (items 10 and 23)
- Positive reframing (items 12 and 17)
- Planning (items 14 and 25)

Each subscale ranges from 2 to 8, and the total score for “problem-focused coping” ranges from 8 to 32.

##### 3) “Emotion-focused coping” domain (items 5, 9, 13, 15, 18, 20, 21, 22, 24, 26, 27, and 28)

This domain represented strategies used to manage emotional distress rather than the stressor itself. It included six subscales:

- Emotional support (items 5 and 15)
- Venting (items 9 and 21)
- Humor (items 18 and 28)
- Acceptance (items 20 and 24)
- Religion (items 22 and 27)
- Self-blame (items 13 and 26)

Each subscale ranges from 2 to 8, and the total score for “emotion-focused coping” ranges from 12 to 48.

##### 4) “Avoidant coping” domain (items 1, 3, 4, 6, 8, 11, 16, and 19)

This domain reflected disengagement or avoidance of the stressor and included four subscales:

- Self-distraction (items 1 and 19)
- Denial (items 3 and 8)
- Substance use (items 4 and 11)
- Behavioral disengagement (items 6 and 16)

Each subscale ranges from 2 to 8, and the total score for “Avoidant Coping” ranges from 8 to 32.

In the present study, items 4 and 11 (substance use) were removed, as they were not suitable for the Bahraini context (due to references to alcohol use, which is taboo among the studied population). Consequently, the total number of items used was 26, and the substance use subscale was excluded from scoring. After this adjustment, the “avoidant coping” domain included three subscales (self-distraction, denial, and behavioral disengagement), with a revised total score range of 6 to 24. The “problem-focused coping” and “emotion-focused coping” domains remained unchanged, with total score ranges of 8–32 and 12–48, respectively.

##### 5) Scoring

To calculate the scores, the responses for the two items in each subscale were summed to obtain the subscale total. The sum of all subscales within each domain produced the total score for that coping domain. Higher scores indicated a greater tendency to use that specific coping approach.

The Brief-COPE does not have standardized cutoff points; therefore, scores were interpreted by comparing mean values across domains or participant groups. Generally, higher “problem-focused coping” scores suggested adaptive and constructive coping behaviors, higher “emotion-focused coping” scores reflected emotional regulation and acceptance strategies, while higher “avoidant coping” scores indicated maladaptive strategies, such as denial or disengagement.

#### Tool III: Ten-Item Depression Anxiety Stress Scale (DASS-10)

##### 1) Description

The original “Depression, Anxiety, and Stress Scale” (DASS) was developed by Lovibond and colleagues [16] to provide a comprehensive assessment of three key dimensions of psychological distress: depression, anxiety, and stress. The DASS-10 is a shortened version, designed to offer a concise yet effective evaluation of these dimensions, making it widely applicable in both clinical and research settings. The original DASS consists of 11 items, with a total score ranging from 0 to 30. Higher scores indicate a greater number or severity of symptoms. However, item 11 was excluded from scoring in this study, as all participants responded with “never,” indicating no variability in responses.

##### 2) Subscales

The DASS-10 was divided into two subscales:

- Anxiety–stress subscale: items 1, 4, 6, 7, 8, and 9 (raw score range = 0 to 18)
- Depression subscale: items 2, 3, 5, and 10 (raw score range = 0 to 12)

Each item is rated on a four-point Likert-type scale (0 = “not at all,” 1 = “some of the time,” 2 = “considerable degree,” and 3 = “very much”).

##### 3) Scoring

In addition to raw scores, average scores were calculated by dividing the raw score by the number of items in each subscale. This approach provides a clearer understanding of the respondent’s general pattern of responses and allows for meaningful comparisons between subscales and the total score. Severity levels were classified as follows:

- Mild/subclinical: raw score ≤ 6, average score ≤ 0.6 (≤ 83rd percentile)
- Moderate: raw score 7–12, average score 0.7–1.2 (84th–99.8th percentile)
- Severe: raw score ≥ 13, average score 1.3–3.0 (≥ 99.9th percentile)

A normative percentile was computed based on a community sample [17], indicating how the respondent’s score compares to the general adult population. For example, a percentile of 83 or less suggested that the individual experiences less distress than 83% of the population, placing them in the mild/subclinical category. In mental health settings, it was common to observe percentiles in the 90s.

When administered multiple times, average scores could be graphed to illustrate changes in symptoms over time. Based on reliable change calculations, the following interpretations were used to describe symptom changes from the first to the most recent administration:

- Deterioration: increase in scores by 5 or more.
- No reliable change: score change of 4 or less.
- Reliable improvement: decrease in scores by 5 or more.
- Recovery: decrease in scores by 5 or more, with the most recent score falling within the mild/subclinical range (score ≤ 6).

### Data collection/procedure

During the data collection procedure, the researcher attended oncology clinics in governmental hospitals from June 2023 to December 2023. Printed copies of the three research questionnaires were distributed to eligible survivors who met the inclusion criteria for the study.

The researcher was present at the clinics during working hours to explain the purpose of the study, answer any questions, and provide assistance as needed, ensuring that participants completed the questionnaires independently and in a comfortable environment.

Completed questionnaires were collected on a daily basis, to maintain data integrity and confidentiality. All collected data were initially entered into Microsoft Excel for organization and preliminary review. Subsequently, the data were transferred to SPSS for statistical analysis with the assistance of the statistician.

Descriptive and inferential statistical analyses were performed to examine participants’ responses. As described above, the instruments used in this study included a demographic information questionnaire, the Brief-COPE questionnaire, and the DASS-10. Data were analyzed using SPSS software (version 28.0).

### Ethical considerations

Approval for conducting the study was obtained from the Ethical Research Review Board of the College of Health and Sport Sciences (The Scientific Research and Publication Committee CHSS SRPC, No. 58/2022-23), followed by permission from governmental hospitals’ Research and Ethics committees (No. 71150623). Written informed consent was obtained from the participants after explaining the aim and objectives of the study. Participation in the study was entirely voluntary. The right to refuse, participate, or withdraw from the study was emphasized. Confidentiality of the data obtained was assured, and participants’ anonymity was respected.

## Results

### Frequency and percentage distribution of demographic characteristics of the participants (N = 104)

Table 2 presents the sociodemographic and clinical characteristics of the study participants, comprising a total of 104 individuals diagnosed with BC who met the inclusion criteria. Approximately half of the participants were aged 50–59, while about a third were aged 40–49 years. The majority of participants were married (n = 70, 67.3%), while nearly a quarter were widowed or divorced (n = 24, 23.1%), and approximately a tenth were single (n = 10, 9.6%).

**Table 2:** Frequency and Percentage Distribution of Demographic Characteristics of Participants (N = 104)

Characteristics	n (%)
Age (years)	
< 40	12 (11.5)
40–49	36 (34.6)
50+	56 (53.9)
Marital status	
Single	10 (9.6)
Married	70 (67.3)
Widowed/ divorced	24 (23.1)
Level of education	
Uneducated	19 (18.3)
High school or below	50 (48.1)
University or higher	35 (33.7)
Employment status	
Employed	10 (9.6)
Unemployed	57 (54.8)
Retired	37 (35.6)
Comorbidities	
Yes	71 (68.3)
No	33 (31.7)
Surgeries related to the disease (n = 104)	
Complete breast removal with axillary lymph nodes right side	37 (35.6)
Complete breast removal with axillary lymph nodes left side	55 (52.9)
Complete breast removal with axillary lymph nodes both sides	12 (11.5)

Source: Authors’ original creation.

Regarding their educational level, nearly half of the participants cited high school qualifications or below (n = 50, 48.1%), while about a third held university degrees or higher (n = 35, 33.7%). However, almost a fifth of the sample had no formal education (n = 19, 18.3%). In terms of employment status, more than half of the participants were unemployed (n = 57, 54.8%), while 35.6% were retired (n = 37). Only 9.6% were currently employed (n = 10).

With respect to health conditions, only 32% of the participants reported no comorbidities (n = 33), whereas the majority (n = 71, 68.3%) had one or more comorbid conditions. Approximately half of the participants exhibit complete breast removal with axillary lymph node dissection on the left side (n = 55, 52.9%).

### Brief-COPE

Table 3 shows the mean and standard deviation (SD) values for participants’ scores on the Brief-COPE tool with its three subscales, including “problem-focused coping,” “emotion-focused coping,” and “avoidant coping.” The analysis of C-Strats among the participants reveals distinct patterns in how they manage stress. The coping mechanisms are categorized into three main subscales, each showing varying levels of use based on the participants’ responses. The results show that “problem-focused coping” (M = 2.28, SD = 0.69) is the most frequently used coping method, follow by “emotion-focused coping” (M = 2.10, SD = 0.33). The least adopted coping mechanisms are avoidant C-Strats (M = 1.82, SD = 0.39).

**Table 3:** Mean and Standard Deviation for Brief-COPE (N = 104)

Dimension	Mean ± SD
1. “Problem-focused coping”	2.28 ± 0.69
Active coping	
2. “I have been concentrating my efforts on doing something about the situation I’m in.”	2.38 ± 0.96
7. “I have been taking action to try to make the situation better.”	2.41 ± 0.92
Subtotal	2.39 ± 0.84
Use of informational support	
10. “I have been getting help and advice from other people.”	2.59 ± 1.01
23. “I have been trying to get advice or help from other people about what to do.”	2.57 ± 0.9
Subtotal	2.58 ± 0.79
Positive reframing	
12. “I have been trying to see it in a different light, to make it seem more positive.”	2.25 ± 0.88
17. “I have been looking for something good in what is happening.”	2.15 ± 0.81
Subtotal	2.2 ± 0.75
Planning	
14. “I have been trying to come up with a strategy about what to do.”	1.98 ± 0.9
25. “I have been thinking hard about what steps to take.”	1.92 ± 0.95
Subtotal	1.95 ± 0.82
2. “Emotion-focused coping”	2.10 ± 0.33
Emotional support	
5. “I have been getting emotional support from others.”	2.75 ± 0.84
15. “I have been getting comfort and understanding from someone.”	2.25 ± 0.98
Subtotal	2.5 ± 0.77
Venting	
9. “I have been saying things to let my unpleasant feelings escape.”	1.67 ± 0.69
21. “I have been expressing my negative feelings.”	1.84 ± 0.85
Subtotal	1.75 ± 0.58
Humor	
18. “I have been making jokes about it.”	1.2 ± 0.55
28. “I have been making fun of the situation.”	1.02 ± 0.14
Subtotal	1.11 ± 0.28
Acceptance	
20. “I have been accepting the reality of the fact that it has happened.”	2.34 ± 0.66
24. “I have been learning to live with it.”	2.64 ± 0.74
Subtotal	2.49 ± 0.59
Religion	
22. “I have been trying to find comfort in my religion or spiritual beliefs.”	3.38 ± 0.78
27. “I have been praying or meditating.”	3.32 ± 0.86
Subtotal	3.35 ± 0.74
Self-blame	
13. “I have been criticizing myself.”	1.34 ± 0.81
26. “I have been blaming myself for things that happened.”	1.51 ± 0.92
Subtotal	1.42 ± 0.76
3. “Avoidant Coping”	1.82 ± 0.39
Self-distraction	
1. “I have been turning to work or other activities to take my mind off things.”	2.68 ± 0.92
19. “I have been doing something to think about it less, such as going to movies, watching TV, reading, daydreaming, sleeping, or shopping.”	2.63 ± 0.84
Subtotal	2.65 ± 0.76
Denial	
3. “I have been saying to myself ‘this isn’t real.’”	1.52 ± 0.75
8. “I have been refusing to believe that it has happened.”	1.42 ± 0.65
Subtotal	1.47 ± 0.58
Behavioral disengagement	
6. “I have been giving up trying to deal with it.”	1.3 ± 0.62
16. “I have been giving up the attempt to cope.”	1.38 ± 0.78
Subtotal	1.34 ± 0.58

Source: Authors’ original creation. Items from Brief-COPE [15].

For “problem-focused coping” (M = 2.28, SD = 0.69), participants moderately rely on strategies aim at addressing problems directly. The most common approaches include active coping (M = 2.39, SD = 0.84), whereby participants take unhurried action to improve the situation, and seeking informational support (M = 2.58, SD = 0.79), indicating that they often turn to others for advice and guidance. However, positive reframing (M = 2.20, SD = 0.75), or attempting to view the situation more positively, and planning (M = 1.95, SD = 0.82) are used less frequently, suggesting some challenges in maintaining optimism or developing concrete strategies for problem-solving.

For “emotion-focused coping” (M = 2.10, SD = 0.33), participants commonly search for emotional support (M = 2.50, SD = 0.77), relying on others for comfort and understanding, reflecting the importance of social connections during stressful periods. Acceptance (M = 2.49, SD = 0.59), or coming to terms with the situation, was also frequently used. The highest mean score across all items is observed for religion (M = 3.35, SD = 0.74), indicating that many participants found strength and comfort through their religious or spiritual beliefs. Conversely, venting (M = 1.75, SD = 0.58), humor (M = 1.11, SD = 0.28), and self-blame (M = 1.42, SD = 0.76) are less commonly used, suggesting that participants are less likely to cope by expressing frustration, using humor, or blaming themselves.

For “avoidant coping” (M = 1.82, SD = 0.39), self-distraction (M = 2.65, SD = 0.76) is a moderately common strategy, as participants often turn to various activities, such as work or entertainment, to divert their attention from stress. In contrast, denial (M = 1.47, SD = 0.58), or refusal to acknowledge the reality of the situation, and behavioral disengagement (M = 1.34, SD = 0.58), which involves giving up attempts to cope, are less frequently used.

Overall, Table 4 demonstrates that participants use a variety of C-Strats, with higher reliance on social and spiritual support, moderate use of problem-solving approaches, and minimal use of avoidant or negative coping mechanisms such as self-blame or denial. Among all C-Strats, religion emerges as the most commonly us coping mechanism (M = 3.35, SD = 0.74), while humor was the least used (M = 1.11, SD = 0.28).

**Table 4:** Overall Total Mean and Standard Deviation for Brief Coping Strategies

Subscales for coping	Overall total, mean ± SD
“Problem-focused coping”	2.2 ± 0.75
“Emotion-focused coping”	1.82 ± 0.39
“Avoidant coping”	1.34 ± 0.58
Total	1.17 ± 0.69

Source: Authors’ original creation. Scales from Brief-COPE [15].

### DASS-10

Table 5 illustrates that women diagnosed with BC experience moderate levels of anxiety, and mild to moderate depressive symptoms. Anxiety items’ mean scores range from 2.31 to 3.13, with an overall mean of approximately 2.54. These values indicate that while participants did not report overwhelming anxiety, they frequently encounter feelings of fear without identifiable causes. The highest score (M: 3.13) corresponds to unexpected fears, whereas the lowest score (M: 2.31) reflects concerns about potential panic situations. SDs range from 0.77 to 0.88, suggesting variability in individual responses.

**Table 5:** Mean and Standard Deviation of DASS-10 (N = 104)

	Mean ± SD
Anxiety–stress	
1. “I felt I was close to panic.”	2.91 ± 0.80
4. “I was intolerant of anything that kept me from getting on with what I was doing.”	2.47 ± 0.77
6. “I felt scared without any good reason.”	3.13 ± 0.78
7. “I tend to overreact to situations.”	2.34 ± 0.85
8. “I was worried about situations in which I might panic and make a fool of myself.”	2.31 ± 0.83
9. “I found it difficult to relax.”	2.78 ± 0.88
Subtotal	2.63 ± 0.25
Depression	
2. “I found it difficult to work up the initiative to do things.”	2.68 ± 0.74
3. “I felt down-hearted and blue.”	2.82 ± 0.80
5. “I felt that I have nothing to look forward to.”	2.41 ± 0.79
10. “I couldn’t seem to experience any positive feelings at all.”	2.28 ± 0.78
Subtotal	2.55 ± 0.24

Source: Authors’ original creation. Scales from DASS-10 [16].

Mean depression scores range from 2.28 to 2.82, with an overall mean of 2.55. These findings imply that participants struggle with sadness and reduced motivation. The highest score (M: 2.82) is associated with feelings of being downhearted, while the lowest score (M: 2.28) pertains to difficulties in experiencing positive emotions. SDs (0.74 to 0.80) further underscore the diversity of emotional experiences among the participants.

Table 6 confirms moderate levels of psychological distress. The “anxiety–stress” mean score was 2.63 (SD: 0.25), indicating a consistent pattern of notable anxiety and stress. Similarly, the “depression” mean score was 2.55 (SD: 0.24), reflecting comparable levels of depressive symptoms. The overall mean score across both categories was 2.59 (SD: 0.25), reinforcing the presence of emotional challenges within the study population.

**Table 6:** Summary of Descriptive and Correlation Interpretation between Anxiety and Depression (N= 104)

DASS-10 subscales	Total, mean ± SD	Interpretation
Anxiety–stress	2.63 ± 0.25	Moderate level of anxiety-stress
Depression	2.55 ± 0.24	Moderate level of depressive symptoms
Total	2.59 ± 0.25	

Source: Authors’ original creation.

These findings underscore the importance of mental health awareness and the implementation of tailor interventions to support BC patients, many of whom could benefit from target strategies to address their specific psychological needs.

A Pearson correlation analysis to examine the relationship between anxiety–stress and depression scores indicates a strong positive correlation [17], suggesting that participants with higher anxiety–stress levels also report greater depressive symptoms (r = 0.68, P < 0.001).

### Associations with demographic variables

The present study investigated differences in C-Strats (Brief-COPE) and Psych-WB (DASS-10) among survivors according to selected demographic characteristics in Table 7.

**Table 7:** Differences in Mean Scores of Copings (Brief-COPE) and Psychological Well-Being (DASS-10) by Demographic Characteristics (N = 104)

Characteristics	Brief-COPE (1–4), mean ± SD	DASS-10 (1–4), mean ± SD
Age		
< 40 years	2.12 ± 0.37	2.63 ± 0.25
40–49 years	2.13 ± 0.38	2.63 ± 0.25
≥ 50 years	1.99 ± 0.41	2.55 ± 0.24
P value	0.448	0.035
Marital status		
Single	1.98 ± 0.48	2.55 ± 0.30
Married	2.13 ± 0.35	2.45 ± 0.25
Widow/divorced	2.04 ± 0.46	2.60 ± 0.35
P value	0.391	0.071
Level of education		
Uneducated	1.98 ± 0.46	2.70 ± 0.30
High school or below	2.08 ± 0.41	2.50 ± 0.25
University or higher	2.17 ± 0.30	2.40 ± 0.20
P value	0.198	0.088
Employment status		
Employed	2.12 ± 0.37	2.40 ± 0.30
Unemployed	2.08 ± 0.39	2.75 ± 0.25
Retired	2.10 ± 0.40	2.50 ± 0.20
P value	0.940	0.075
Comorbidities		
Yes	2.07 ± 0.42	2.80 ± 0.35
No	2.15 ± 0.33	2.50 ± 0.30
P value	0.334	0.940
Type of surgery		
Right side	2.08 ± 0.38	2.70 ± 0.28
Left side	2.05 ± 0.34	2.90 ± 0.32
Both sides	2.35 ± 0.59	2.45 ± 0.25
P value	0.061	0.257

Source: Authors’ original creation.

Regarding C-Strats, the findings revealed no statistically significant differences across all demographic variables, including age, marital status, education level, employment status, comorbidities, and type of surgery (P > 0.05). Although slight variations were observed—for instance, higher coping scores among participants with higher education levels and those who underwent bilateral surgery—these differences did not reach statistical significance. This suggests that C-Strats may be relatively stable across different demographic groups within this population and are potentially influenced by other factors not examined in this study.

In contrast, Psych-WB (DASS-10) demonstrated a statistically significant difference in relation to age (P = 0.035). Younger survivors (aged below 50 years) reported higher levels of psychological distress compared to older participants. This finding may indicate that younger individuals experience greater emotional and psychological burden, possibly due to added life stressors such as family responsibilities, career concerns, and future uncertainty following diagnosis and treatment.

Although not statistically significant, trends were observed across other variables. Higher distress levels were noted among unemployed participants and those with lower educational levels, suggesting a potential influence of socioeconomic factors on Psych-WB. Similarly, participants with comorbid conditions and those who had left-side surgery reported slightly higher distress scores, though these differences were not significant.

Overall, the findings indicate that while C-Strats did not vary significantly across demographic groups, Psych-WB was influenced by age, with younger survivors being more vulnerable to distress. These results highlight the importance of targeted psychological support interventions, particularly for younger survivors, to enhance their mental health outcomes.

### C-Strats and Psych-WB

The present study examined the relationship between C-Strats (Brief-COPE) and Psych-WB (DASS-10) among survivors (Table 8).

**Table 8:** Pearson Correlation Between Coping (Brief-COPE) and Psychological Well-Being (DASS-10) (N = 104)

Variables	Brief-COPE	DASS-10
Brief-COPE		
Correlation	1	
P value		
DASS-10		
Correlation	−0.501**	
P value	< 0.001	1

**Correlation is significant at the 0.01 level. Source: Authors’ original creation.

The findings revealed a moderate, statistically significant negative correlation between C-Strats and psychological distress (r = −0.501, P < 0.001). This indicates that higher levels of coping are associated with lower levels of psychological distress among the participants. In other words, individuals who reported more effective C-Strats tended to experience better Psych-WB.

The strength of this relationship (moderate correlation) suggests that C-Strats play an important role in influencing the psychological state of survivors, although they are not the only contributing factor. These results highlight the protective role of adaptive coping mechanisms in reducing symptoms of stress, anxiety, and depression.

Overall, the findings emphasize the importance of promoting effective C-Strats as part of supportive care interventions aimed at improving Psych-WB in survivors.

## Discussion

This research explored links among C-Strats and Psych-WB among a sample of Bahrain’s survivors, representing a historically marginalized and disadvantaged group, in addition to examining differences across demographic characteristics. The findings provide important insights into psychosocial adjustment in this population.

### C-Strats and demographic characteristics

The results indicated that C-Strats were not significantly associated with any of the examined demographic variables, including age, marital status, education, employment, comorbidities, and type of surgery. This finding suggests that coping behaviors may be relatively consistent across demographic groups within this sample.

This result is consistent with previous research indicating that coping is influenced more by individual psychological processes, cognitive appraisal, and emotional regulation rather than demographic characteristics alone [18, 19]. However, other studies have reported conflicting findings, where higher education and employment were associated with more adaptive coping due to increased access to resources and health literacy [20]. The discrepancy may be attributed to cultural context, as shared healthcare systems and social structures in Bahrain may reduce socioeconomic differences in coping [5].

### Psych-WB and demographic characteristics

In contrast to coping, Psych-WB (DASS-10) showed a significant relationship with age, with younger participants reporting higher levels of distress. This finding aligns with previous literature indicating that younger survivors are more vulnerable to psychological distress due to factors such as disrupted life plans, fertility concerns, body image issues, and caregiving responsibilities [19, 20]. Although not statistically significant, trends suggested higher distress among participants with lower education, unemployment, and comorbidities. These findings are supported by previous studies demonstrating that socioeconomic disadvantage is associated with increased psychological distress in cancer survivors [19]. Additionally, unemployment following cancer diagnosis has been linked to financial strain and poorer psychosocial outcomes [21]. The lack of statistical significance in the current study may reflect the relatively small sample size or limited variability among participants.

### Relationship between C-Strats and Psych-WB

A key finding of this study was the significant negative correlation between C-Strats and psychological distress (r = −0.501, P < 0.001), indicating that higher coping ability is associated with lower distress levels. This result is consistent with a large body of literature highlighting the protective role of coping in cancer adjustment.

Meta-analytic evidence has shown that higher coping self-efficacy is strongly associated with lower distress and better QoL among cancer patients [21]. Similarly, studies have demonstrated that adaptive C-Strats, such as problem-focused and positive coping, are linked to improved psychological outcomes, while “avoidant coping” is associated with increased distress [22]. Conversely, some studies suggest that the relationship between coping and psychological outcomes may vary depending on the type of C-Strat employed, as well as contextual factors such as disease stage and social support [23]. Since the current study assessed coping as a global construct, it may not capture differences between adaptive and maladaptive coping, which could explain variability in findings across studies.

### Clinical implications

The present findings highlight the importance of integrating psychosocial care into oncology services. Given that C-Strats are significantly associated with Psych-WB, interventions aimed at enhancing adaptive coping skills—such as cognitive behavioral therapy, stress management, and resilience training—may improve mental health outcomes among survivors. Previous evidence supports the effectiveness of such interventions in reducing distress and improving QoL in oncology populations [24–26]. Furthermore, the higher levels of distress observed among younger survivors underscore the need for age-specific psychological support programs tailored to their unique challenges.

### Limitations

The most significant shortcoming of this research is its snapshot view. The cross-sectional design employed undermines the scope to infer causality in identified suggestive relations among C-Strats and Psych-WB. Second, the relatively small sample size (N = 104) may have reduced statistical power, particularly in detecting differences across demographic variables. Third, the use of self-reported measures may introduce response bias, including social desirability bias. Fourth, the study assessed C-Strats as a general construct, without differentiating between adaptive and maladaptive coping, which may limit the depth of interpretation.

Additionally, this research specifically honed in on a particular cultural context (Bahrain); while this was a deliberate decision, to focus on an underserved and disadvantaged population, it nevertheless which inherently reduces generalizability.

### Recommendations

Based on the study findings, several recommendations can be proposed.

#### Clinical practice

- Integrate routine psychological screening (e.g., DASS-10) into oncology care.
- Develop psychosocial interventions focused on strengthening adaptive C-Strats.
- Provide targeted psychological support for younger survivors.

#### Education and training

- Train healthcare professionals in psychosocial oncology and coping-based interventions.
- Increase awareness of the importance of coping in improving psychological outcomes.

#### Future research

- Conduct longitudinal studies to examine causal relationships between coping and Psych-WB.
- Explore different types of C-Strats (e.g., problem-focused vs. emotion-focused).
- Increase sample size and include diverse populations to improve generalizability.

### Conclusions

The outcomes of this study, which offers seminal insights into the experiences of a historically marginalized and underserved patient population (i.e., women in Bahrain) [5], demonstrate that C-Strats are significantly associated with Psych-WB among survivors, with higher coping linked to lower levels of distress. While coping did not differ significantly across demographic characteristics, younger survivors were more likely to experience psychological distress. These findings emphasize the critical role of coping in cancer survivorship and highlight the need for targeted psychosocial interventions to improve mental health outcomes, particularly among vulnerable groups.

## Data Availability

The data supporting the findings of this study are available from the corresponding author upon reasonable request.

## Abbreviations

BC: breast cancer
Brief-COPE: Brief Coping Orientation to Problems Experienced
COPE: Coping Orientation to Problems Experienced
C-Strats: coping strategies
DASS: Depression, Anxiety, and Stress Scale
DASS-10: Ten-Item Depression Anxiety Stress Scale
GCC: Gulf Cooperation Council
Psych-WB: psychological well-being
QoL: quality of life
SD: standard deviation
